# Fluorescent Ureteral Catheter–Assisted Laparoscopic Nephrectomy for a Pediatric Patient With Obstructed Hemivagina and Ipsilateral Renal Anomaly Syndrome

**DOI:** 10.1155/criu/9979542

**Published:** 2026-05-21

**Authors:** Masaki Shinohara, Kenichiro Konishi, Shigemitsu Kojima, Hisashi Kanda, Takeshi Yamaguchi, Akira Nishi

**Affiliations:** ^1^ Department of Surgery, Gunma Children′s Medical Center, Shibukawa, Gunma, Japan

**Keywords:** ectopic atrophic kidney, fluorescent ureteral catheter, laparoscopic nephrectomy, obstructed hemivagina and ipsilateral renal anomaly syndrome

## Abstract

Obstructed hemivagina and ipsilateral renal anomaly (OHVIRA) syndrome is a rare congenital disorder characterized by Müllerian duct malformation and ipsilateral renal abnormalities. Variant forms associated with hypoplastic kidneys and ectopic ureteral insertion in the vagina may require nephrectomy because of persistent urinary incontinence. In such cases, urinary drainage through a vaginal opening permits direct ureteral access via vaginoscopy without the size limitations associated with transurethral cystoscopy. Therefore, retrograde catheter placement is technically feasible in patients with OHVIRA syndrome. Consequently, OHVIRA is a particularly suitable indication for ureteral catheterization. Fluorescent ureteral catheters, which were recently introduced in surgery for adults, enable real‐time intraoperative visualization of the ureter and may further enhance surgical safety. We report a case involving laparoscopic nephrectomy for OHVIRA syndrome with an ectopic ureter and severely hypoplastic kidney in a pediatric patient. The ectopic ureteral orifice was successfully identified during vaginoscopy when the patient was 5 years of age; therefore, retrograde placement of a fluorescent ureteral catheter was feasible. Intraoperative fluorescence enabled the reliable identification of both the ureter and small renal remnant, thus facilitating safe dissection and resection. This case demonstrates that retrograde placement of a fluorescent ureteral catheter via vaginoscopy is feasible and useful for pediatric patients with OHVIRA syndrome who require nephrectomy. This technique may improve intraoperative ureteral visualization and surgical safety; therefore, it should be considered for selected patients with ectopic ureteral drainage.

## 1. Introduction

Obstructed hemivagina and ipsilateral renal anomaly (OHVIRA) syndrome is a rare congenital disorder characterized by Müllerian duct malformations with associated ipsilateral renal abnormalities [1]. Variant forms of OHVIRA involving hypoplastic kidneys and ectopic ureteral insertion in the vagina can occur; in these cases, continuous urinary incontinence is a typical clinical presentation, and nephrectomy may be required when the affected kidney is dysplastic and nonfunctional [2–4].

Urinary drainage through a vaginal opening allows direct ureteral access via vaginoscopy without size limitations associated with cystoscopy in infants and small children. Because of this unique anatomical condition, retrograde catheter placement is technically feasible in nearly all cases of OHVIRA that require nephrectomy.

Retrograde catheterization is particularly valuable in pediatric laparoscopic nephrectomy because the small ureteral caliber, relatively limited retroperitoneal fat, and poorly developed anatomical planes compared with those in adults result in ureteral identification challenges [5, 6]. Although conventional ureteral catheters can assist in ureteral identification through tactile or visual tracing, they do not provide continuous or objective guidance. In contrast, fluorescent ureteral catheters, which were recently introduced in surgery for adults, enable real‐time ureteral visualization under near‐infrared imaging and may reduce the risk of intraoperative misidentification [7–9]. However, to our knowledge, the use of fluorescent ureteral catheters in nephrectomy for pediatric patients has not yet been reported.

These challenges are further exacerbated by conditions associated with ectopic ureters and severely atrophic renal remnants, such as OHVIRA syndrome, that comprise atypical ureteral courses and minimal renal tissue, which often complicate intraoperative identification [10]. Therefore, we report a case of OHVIRA in a pediatric patient that was safely treated with laparoscopic nephrectomy using a fluorescent ureteral catheter and propose OHVIRA syndrome as a suitable indication for this technique.

## 2. Case Report

**Figure 1:**
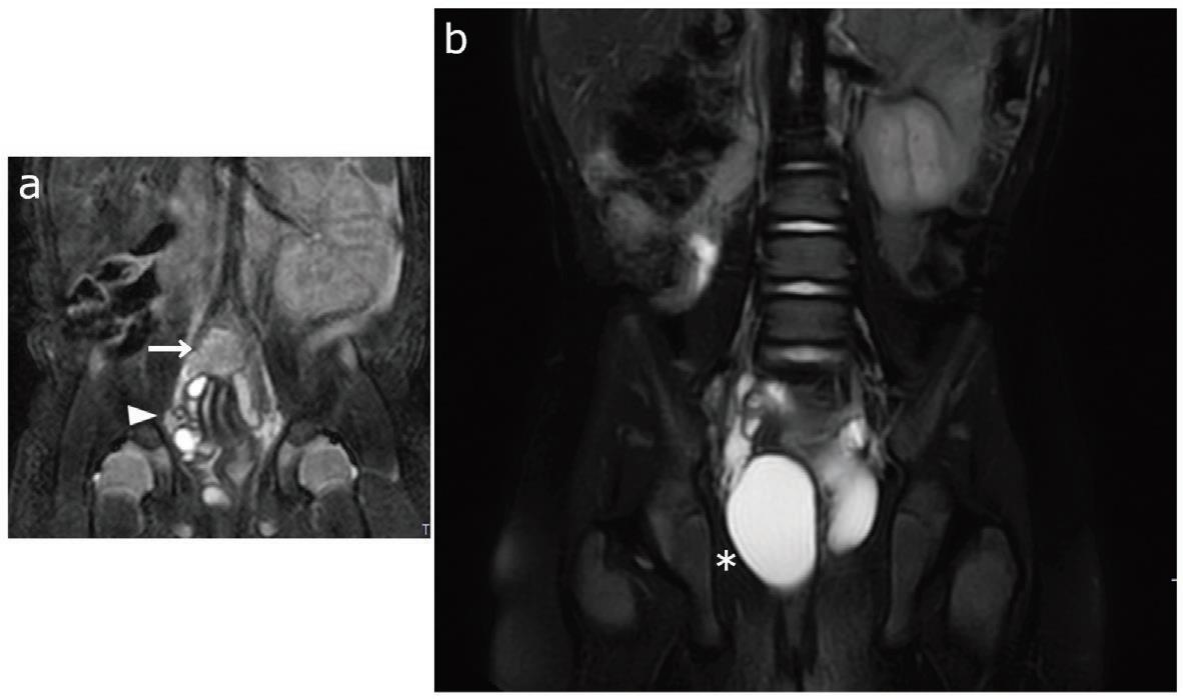
(a) Neonatal magnetic resonance imaging shows a hypoplastic right kidney (arrow) and a small tubular structure consistent with a ureter (arrowhead). (b) During follow‐up, the kidney gradually became indistinct; however, progressive dilation of the right obstructed hemivagina was observed at 1 year and 8 months of age (asterisk). Based on these findings, obstructed hemivagina and ipsilateral renal anomaly syndrome was diagnosed.

Right renal agenesis was prenatally suspected in a female patient (age: 5 years and 8 months) without developmental disorders (height: 111 cm; weight: 17.4 kg; body mass index: 14.1 kg/m^2^). Neonatal magnetic resonance imaging (MRI) revealed a hypoplastic right kidney and a small tubular structure consistent with a ureter (Figure 1a). During follow‐up, the renal remnant gradually became indistinct; however, dilation of the right obstructed hemivagina increased (Figure 1b). Therefore, OHVIRA syndrome was diagnosed.

Because ultrasonography indicated that vaginal dilation remained stable, intervention was deferred to allow for safer manipulation at an older age. At 5 years and 1 month of age, fenestration of the obstructed hemivagina was performed under cystoscopic and vaginoscopic guidance. A bladder evaluation using a pediatric diagnostic cystoscope with a 7.9‐Fr outer sheath without a working channel (A37000A; Olympus, Tokyo, Japan) indicated no identifiable right ureteral orifice. Vaginoscopy using the same scope demonstrated a fenestrated but edematous obstructed hemivagina, which limited visualization and precluded the identification of an ectopic ureteral opening.

The patient had completed toilet training and had no history of urinary incontinence prior to surgery. However, urinary incontinence developed immediately after discharge following fenestration of the obstructed hemivagina and was confirmed at the first postoperative outpatient visit 1 week later. The incontinence was significant enough to require the use of absorbent pads during daytime activities. Given the abrupt onset and severity of symptoms, the incontinence was considered unlikely to be attributable to previously unrecognized sphincter dysfunction. Instead, it was presumed to be associated with ectopic ureteral drainage into the fenestrated hemivagina, which became clinically apparent after fenestration.

Further evaluation was conducted, and technetium‐99m dimercaptosuccinic acid scintigraphy revealed the absence of right kidney function. MRI and ultrasonography revealed a 14‐mm atrophic kidney located cranial to the bladder; however, the ureter could not be visualized. Because of the extremely small renal remnant and anticipated atypical ureteral course, repeat vaginoscopy and planned retrograde placement of a fluorescent ureteral catheter were performed.

**Figure 2:**
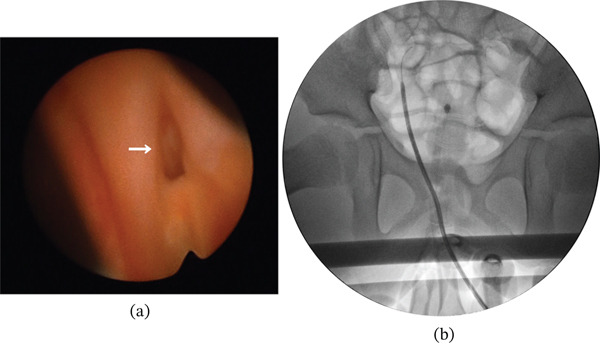
(a) Repeat vaginoscopy image showing improved mucosal edema following fenestration and decompression, thus enabling clear visualization of an ectopic ureteral orifice adjacent to the fenestration site (arrow). (b) Retrograde insertion of a 6‐Fr fluorescent ureteral catheter under fluoroscopic guidance.

Repeat vaginoscopy revealed improved mucosal edema after fenestration and decompression, thus allowing clear visualization of an ectopic ureteral orifice adjacent to the fenestration site (Figure 2a). Retrograde insertion of a 6‐Fr fluorescent ureteral catheter was performed using a 9.5‐Fr outer sheath with a 3‐Fr working channel (A37001A; Olympus, Tokyo, Japan) and guidewire under fluoroscopic guidance (Figure 2b). The catheter is composed of polyurethane with an embedded fluorescent dye; although the exact chemical composition of the fluorophore is proprietary and not publicly disclosed, it exhibits near‐infrared fluorescence under an excitation wavelength of 750–810 nm. The fluorescence characteristics are distinct from those of indocyanine green, although they demonstrate similar optical properties. The fluorescent signal was visualized intraoperatively using a near‐infrared imaging system (1688 AIM 4 K camera system with an L11 light source; Stryker, Kalamazoo, Michigan, United States).

**Figure 3:**
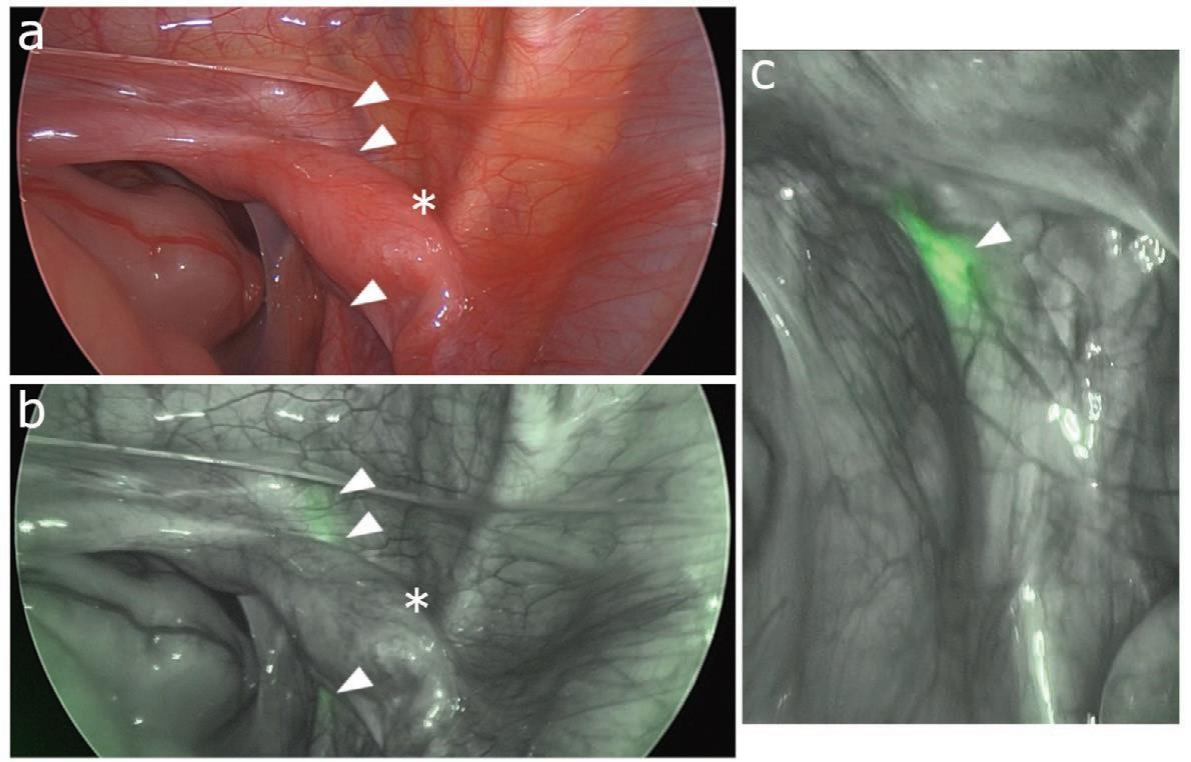
(a, b) During laparoscopy, the ureter coursed posterior to the duplicated uterus (asterisk) and was difficult to distinguish from the surrounding structures; however, fluorescence imaging permitted confident identification of the ureter (arrowheads). (c) Close‐up imaging clearly shows fluorescence of the ectopic ureter containing the fluorescent ureteral catheter (arrowhead).

During laparoscopy, the ureter was found to course along the right side and pass posterior to the duplicated uterus, an unusual anatomical relationship that made it difficult to distinguish from the surrounding structures. Near‐infrared fluorescence imaging clearly delineated the course of the ureter (Figure 3a–c), allowing its safe taping.

**Figure 4:**
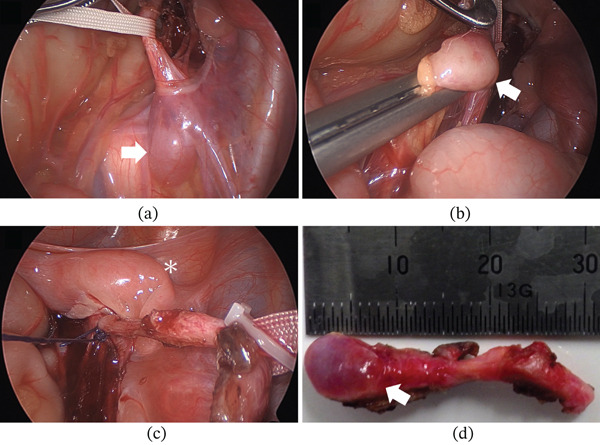
(a) Identification of the atrophic renal remnant (arrow) following cranial dissection after ureteral taping. (b) Division of the renal remnant and its feeding vessel using an energy device (Enseal). (c) Ligation and transection of the ureter at a safely identifiable level without extensive dissection behind the duplicated uterus (asterisk). (d) Resected atrophic kidney measuring approximately 9 mm in diameter (arrow).

After taping, cranial dissection was performed using a combination of blunt dissection and sharp dissection with an energy device (Enseal; Ethicon, Cincinnati, Ohio, United States), leading to identification of the atrophic renal remnant (Figure 4a). The renal remnant was extremely small, consistent with preoperative imaging findings, and had an appearance similar to that of a lymph node. However, as the ureter had been clearly identified using the fluorescent ureteral catheter, the renal remnant could be safely identified without injury to surrounding tissues and with minimal bleeding. A small feeding vessel to the renal remnant was identified and divided using the energy device (Enseal) along with the surrounding tissue (Figure 4b). The fluorescent ureteral catheter was maintained until immediately before ureteral transection to allow real‐time visualization. Fluorescence imaging confirmed that the ureter coursed posterior to the duplicated uterus; therefore, to avoid uterine injury, it was ligated and transected at a safely identifiable level without extensive dissection behind the uterus (Figure 4c). The resected atrophic kidney measured approximately 9 mm in diameter (Figure 4d).

The histopathological examination confirmed atrophic renal parenchyma and a tubular structure consistent with a ureter. The postoperative course was uneventful. Urinary incontinence resolved immediately after nephrectomy, and the patient was discharged on Postoperative Day 4. At the 1‐year postoperative follow‐up, the patient remained asymptomatic, with no recurrence of urinary incontinence or evidence of intra‐abdominal complications, including hematoma, infection, or cystic changes related to the ureteral remnant.

## 3. Discussion

The use of a fluorescent ureteral catheter during laparoscopic nephrectomy enabled safe intraoperative identification of both the ureter and renal remnant in our pediatric patient with OHVIRA syndrome with an ectopic ureter and severely hypoplastic kidney. To our knowledge, this is the first English‐language report of the use of a fluorescent ureteral catheter during pediatric nephrectomy, thus suggesting that this is a novel technical option for pediatric renal surgery.

OHVIRA syndrome is classically characterized by the triad of Müllerian duct anomalies, a duplicated uterus with unilateral obstruction, and ipsilateral renal agenesis [1]. As unilateral vaginal obstruction is an inherent feature of OHVIRA syndrome, hemivaginal fenestration is an essential component of management, as it is required not only for immediate decompression of the obstructed hemivagina but also for the long‐term prevention of complications such as hematometra and hematocolpos as the patient approaches puberty [11, 12]. Although once considered rare, uterovaginal anomalies have been observed in 37%–60% of female patients with renal agenesis, and uterine duplication has been observed in 81% of women [13, 14]. As awareness and diagnostic imaging continue to improve, OHVIRA syndrome detection rates are expected to increase. Several reports have described patients who were initially diagnosed with renal agenesis based on imaging findings but were later found to have atrophic or dysplastic remnants associated with ectopic ureters that resulted in persistent urinary incontinence or vaginal discharge and required nephrectomy [2–4]. These cases highlight the technical challenges posed by atypical ureteral courses and minimal residual renal tissue.

Telecan et al. reported the use of three‐dimensional laparoscopy to enhance visualization and facilitate the identification of the abnormal ureteral course during nephrectomy for renal hypoplasia associated with OHVIRA syndrome [10]. In the present case, the atrophic kidney was only 9 mm and associated with an ectopic ureter; therefore, the identification of both structures without guidance was extremely challenging. The use of fluorescent ureteral catheters enables real‐time intraoperative ureteral visualization, allows reliable visualization of the ureteral course, and facilitates safe taping and resection [5, 6].

Most OHVIRA syndrome cases that require nephrectomy involve ectopic ureteral drainage into the vagina or an obstructed hemivagina [2–4], which is a unique characteristic that is advantageous because vaginoscopic access is less constrained by the age or body size of the patient compared with transurethral cystoscopy. Therefore, retrograde placement of a fluorescent ureteral catheter may be considered for patients with OHVIRA syndrome who require nephrectomy. In male patients, transurethral placement of a 6‐Fr fluorescent ureteral catheter, even when advanced over a guidewire, requires a pediatric cystoscope with a 9.5‐Fr outer sheath and 3‐Fr working channel; thus, it may not be feasible for smaller children with insufficient urethral growth. In contrast, patients with OHVIRA syndrome can undergo vaginoscopic observation and manipulation using the same instruments, even during early childhood. OHVIRA syndrome may thus be a favorable indication for fluorescence‐guided ureteral identification. In this case, the ectopic ureteral orifice was successfully identified using vaginoscopy at 5 years of age; therefore, retrograde placement of a fluorescent ureteral catheter was feasible. Based on this experience, vaginoscopic evaluation may be a useful diagnostic approach in patients with OHVIRA syndrome who require nephrectomy, particularly when an ectopic ureteral opening is suspected. In addition, fluorescence‐guided ureteral catheterization may be considered as a helpful adjunct for intraoperative ureteral identification in selected cases where the ureteral orifice can be identified.

Additionally, in our case, an ectopic ureter was suspected because of urinary incontinence following fenestration of the obstructed hemivagina; however, the orifice was not identified during the initial cystoscopic and vaginoscopic examinations. Ectopic ureteral insertion should be suspected when girls with otherwise normal voiding experience continuous urinary leakage or recurrent urinary tract infections. Diagnostic modalities include ultrasonography, voiding cystourethrography, renal scintigraphy, contrast‐enhanced computed tomography, and MRI; additionally, cystoscopic and vaginoscopic examinations are recommended to determine the definitive diagnosis [15]. Previous reports have suggested that vaginoscopy combined with high‐pressure vaginography may be more effective than computed tomography or MRI for identifying ectopic vaginal ureteral openings [16, 17]. In the present case, mucosal edema within the obstructed cavity likely hindered visualization during the initial examination. Repeat vaginoscopy after decompression allowed successful identification of the ectopic orifice. When initial imaging fails to delineate the ureteral course or opening, a staged strategy involving fenestration of the obstructed hemivagina followed by careful observation for urinary incontinence and repeat vaginoscopy after edema resolution may be useful. High‐pressure vaginography may be an adjunct diagnostic tool in selected cases.

Several limitations of fluorescent ureteral catheters should be acknowledged. Currently available fluorescent ureteral catheters have a diameter of 6 Fr, which may limit their use in infants with very small ureters. Additionally, the indications for their use are restricted to cases in which an ectopic ureteral orifice can be identified. When catheter placement is not feasible, alternative strategies such as conventional retrograde ureterography or intraoperative ultrasonography should be considered. Future development of smaller diameter fluorescent catheters and multicenter studies of their utility for OHVIRA syndrome are warranted.

## 4. Conclusion

This case highlights that retrograde placement of a fluorescent ureteral catheter via vaginoscopy is feasible and useful for pediatric patients with OHVIRA syndrome who require nephrectomy. This technique may improve intraoperative ureteral visualization and surgical safety; therefore, it should be considered for selected patients with ectopic ureteral drainage.

## Author Contributions

Masaki Shinohara and Kenichiro Konishi contributed to the conception and design of the study. Masaki Shinohara performed the surgical procedure and drafted the manuscript. Kenichiro Konishi supervised the study and critically revised the manuscript. Shigemitsu Kojima, Hisashi Kanda, and Takeshi Yamaguchi contributed to data collection and interpretation. Akira Nishi provided overall supervision.

## Funding

No funding was received for this manuscript.

## Disclosure

All authors read and approved the final manuscript.

## Consent

Informed consent for the publication of this case has been obtained from the patient′s parents or guardian.

## Conflicts of Interest

The authors declare no conflicts of interest.

## Data Availability

The data that support the findings of this study are available on request from the corresponding author. The data are not publicly available due to privacy or ethical restrictions.

## References

[1] Smith N. A. and Laufer M. R. , Obstructed Hemivagina and Ipsilateral Renal Anomaly (OHVIRA) Syndrome: Management and Follow-Up, Fertility and Sterility. (2007) 87, no. 4, 918–922, 10.1016/j.fertnstert.2006.11.015, 2-s2.0-34147104020, 17320871. 17320871

[2] Schlomer B. , Rodriguez E. , and Baskin L. , Obstructed Hemivagina and Ipsilateral Renal Agenesis (OHVIRA) Syndrome Should Be Redefined as Ipsilateral Renal Anomalies: Cases of Symptomatic Atrophic and Dysplastic Kidney With Ectopic Ureter to Obstructed Hemivagina, Journal of Pediatric Urology. (2015) 11, no. 2, 77.e1–77.e6, 10.1016/j.jpurol.2014.12.004, 2-s2.0-84930413035, 25797857. 25797857

[3] Nakahara Y. , Nakada S. , Hitomi K. , Hanaki S. , Doi K. , Goto T. , and Aoyama K. , Urological Anomalies Associated With Obstructed Hemivagina and Ipsilateral Renal Anomaly (OHVIRA) Syndrome, A Case Series, Journal of Pediatric Surgery Case Reports. (2020) 52, 101358, 10.1016/j.epsc.2019.101358.

[4] Garge S. , Bagga D. , Acharya S. K. , Yadav D. K. , Khan T. R. , Kumar R. , Kumar V. , Kumar S. , Gupta D. , and Prasad A. , Herlyn-Weber-Wunderlich Syndrome With Ectopic Ureter in Prepubertal Female, Journal of Indian Association of Pediatric Surgeons. (2014) 19, no. 2, 103–105, 10.4103/0971-9261.129607, 2-s2.0-84898872151, 24741215. 24741215 PMC3983760

[5] Kim C. and Docimo S. G. , Use of Laparoscopy in Pediatric Urology, Reviews in Urology. (2005) 7, 215–223. 16985833 PMC1477582

[6] Caione P. , Kavoussi L. R. , Micali F. , and Micali S. , Retroperitoneoscopy and Extraperitoneal Laparoscopy in Pediatric and Adult Urology, 2003, Springer Milan, 10.1007/978-88-470-2923-1.

[7] Fujita H. , Kikuchi I. , Nakagawa R. , Katano M. , Nakano E. , Kitayama R. , and Tanaka Y. , Use of a Novel Fluorescent Catheter to Locate the Ureters During Total Laparoscopic Hysterectomy, Journal of Minimally Invasive Gynecology. (2021) 28, no. 7, 1420–1424, 10.1016/j.jmig.2021.04.004, 33887490. 33887490

[8] Ryu S. , Okamoto A. , Nakashima K. , Hara K. , Ishida K. , Ito R. , and Nakabayashi Y. , Ureteral Navigation Using a Fluorescent Ureteral Catheter During Laparoscopic Colorectal Surgery, Surgical Endoscopy. (2021) 35, no. 8, 4882–4889, 10.1007/s00464-021-08538-3, 33978850. 33978850

[9] Kisu I. , Shiraishi T. , Iijima M. , Nakamura K. , Matsuda K. , and Hirao N. , A Novel Near-Infrared Ray Catheter Fluorescent Ureteral Catheter for Preventing Ureteral Injury in Gynecologic Laparoscopic Surgery, Archives of Gynecology and Obstetrics. (2021) 304, no. 2, 283–284, 10.1007/s00404-021-06103-w, 34050789. 34050789

[10] Telecan T. , Capras R. D. , Filip G. A. , Ionutas E. M. , Stanca D. V. , and Crivii C. B. , OHVIRA Syndrome and Ureteral Ectopy Draining in the Ipsilateral Hemiuterus, Diagnosed in the Prepubertal Age Group: Case-Report and Literature Review, Medicina. (2024) 60, no. 12, 10.3390/medicina60121922, 39768804. PMC11678595 39768804

[11] Gholoum S. , Puligandla P. S. , Hui T. , Su W. , Quiros E. , and Laberge J.-M. , Management and Outcome of Patients With Combined Vaginal Septum, Bifid Uterus, and Ipsilateral Renal Agenesis (Herlyn-Werner-Wunderlich Syndrome), Journal of Pediatric Surgery. (2006) 41, no. 5, 987–992, 10.1016/j.jpedsurg.2006.01.021, 2-s2.0-33646135322, 16677898. 16677898

[12] Candiani M. , Vercellini P. , Ferrero-Caroggio C. , Fedele L. , Salvatore S. , and Fedele L. , Conservative Treatment of Herlyn-Werner-Wunderlich Syndrome: Analysis and Long-Term Follow-Up of 51 Cases, European Journal of Obstetrics & Gynecology and Reproductive Biology. (2022) 275, 84–90, 10.1016/j.ejogrb.2022.06.013, 35763966. 35763966

[13] Thompson D. P. and Lynn H. B. , Genital Anomalies Associated With Solitary Kidney, Mayo Clinic Proceedings. (1966) 41, no. 8, 538–548, 10.1016/S0025-6196(25)07942-X. 5945096

[14] Li S. , Qayyum A. , Coakley F. V. , and Hricak H. , Association of Renal Agenesis and Mullerian Duct Anomalies, Journal of Computer Assisted Tomography. (2000) 24, no. 6, 829–834, 10.1097/00004728-200011000-00001, 2-s2.0-0034463703. 11105695

[15] European Association of Urology , EAU Guidelines on Paediatric Urology: Ectopic Ureter, 2026, EAU Guidelines Office.

[16] Zuckerman J. M. , Shekarriz B. , and Upadhyay J. , High Pressure Vaginography to Diagnose Vaginal Ureteral Ectopia in Patients With Continuous Urinary Incontinence, Canadian Journal Of Urology. (2013) 20, no. 1, 6603–6606, 23433129. 23433129

[17] Goidescu I. G. , Nemeti G. , Staicu A. , Surcel M. , Goidescu C. M. , Rotar I. C. , Cruciat G. , and Muresan D. , Prenatal Diagnosis of Vaginal Ectopic Ureter Insertion-Case Outcome and Literature Overview, Diagnostics. (2025) 15, no. 14, 10.3390/diagnostics15141788, 40722537. PMC12293270 40722537

